# Development and Validation of the Win-Win Scale

**DOI:** 10.3389/fpsyg.2021.657015

**Published:** 2021-05-21

**Authors:** Shan Zhang, Xinlei Zang, Feng Zhang

**Affiliations:** Institute of Psychology and Behavior, Henan University, Kaifeng, China

**Keywords:** win-win, scale development, factor analysis, reliability, validity

## Abstract

Accumulating evidence has shown that win-win is necessary for both individuals and the society. This research, including two studies, aimed to develop and validate a measurement of the win-win scale. In the first study, we screened the items by item analysis and extracted common factors using exploratory factor analysis (EFA), thus determining a total of 25 items in the initial scale consisted of five dimensions including integrity, advancement, altruism, harmoniousness, and coordination. In the second study, we used first- and second-order confirmatory factor analysis (CFA) to test the scale’s construct validity. The results indicated a good fit between the five-factor model and the data. Based on our results, we have formed a win-win scale by keeping 16 items from the original project pool.

## Introduction

Research on the balance between what is best for individuals and what is best for the collective ones has long been central to sociology and other social sciences (Simpson and Willer, 2015). As a social value orientation, win-win is mainly reflected in a situation that one actively considers and takes care of others to pursue personal interests. Win-win is the realization of maximizing the interests of both sides, which is a harmonious development with mutual benefits. On the one hand, competition is not a zero-sum game, and the interests of different parties are so intertwined that the development of one party often benefits others; the damage of one party’s interests will often spread to other parties at the same time. By improving the cooperative relationship between interrelated stakeholders, existing resources can be used more effectively or new resources can be developed so as to achieve the effect that one plus one is greater than two; in other words, all parties work together to “make the cake bigger,” so they can get more benefits. We are a community of shared interests, and we need to ensure that spillover effects on each other are positive, not negative. Therefore, win-win means that the common interests can be maximized first, and the individual interests can be better realized. On the other hand, win-win is based on cooperation that is a key factor in sustaining and stabilizing human society (Falk et al., 2003). Although self-interest is a human instinct, human beings tend to pursue morality, fairness, and justice, which can serve as an adaptive strategy to promote social harmony (Forgas et al., 2007). Being too selfish and too altruistic is not the best way to cooperate (Fehr and Fischbacher, 2003). The success will not be sustainable due to lack of the partner’s cooperation, and so the so-called winners do things harmful to others without benefiting themselves. Therefore, individuals also need to think of others to achieve win-win when they seek personal interests, which is conducive to better survival and development of individuals.

Win-win is a typical Chinese cultural value recognized by scholars globally (Hofstede, 1980), which helps to promote good interaction among people. Interpersonal harmony is an important aspect and practice in Chinese traditional culture (Zhang, 1996). For instance, Confucianism advocated “benevolence, righteousness, propriety, wisdom, faith, forgiveness, loyalty, filial piety fraternity.” Each content contains the guidelines for getting along with others, emphasizing collectivism. The importance of win-win cooperation is also reflected in “if two people reach an agreement, they can overcome all difficulties” in *The Book of Changes* (Tze, 2011) and “one is liable to fail, and if there are many people, it is hard to be defeated” in *History as a Mirror* (Sima, 1956). Rooted in Chinese traditional culture, win-win is a value orientation of globalization and a common pursuit of human beings. It has a significant impact on eastern culture and also plays an essential role in human development in other cultural environments. Advocating win-win values can overcome shortsightedness in the world. In fact, such win-win values are needed not only for China but also for other countries or regions. In addition, a large number of studies have shown that modern society may need to reach win-win from the perspective of collectivism (Yang, 1996; Hamamura, 2012; Van de Vliert et al., 2012; Grossmann and Varnum, 2015; Huang et al., 2016; Santos et al., 2017). Therefore, individuals should learn the attitude of “win-win” when facing limited resources and interests, rather than the attitude of “success only, even without using ethical methods” (Jiao and Su, 2019).

At present, most studies related to win-win in psychology were focused on trust, cooperation, and prosocial behavior (Smith, 2015; Duijf, 2018; Zlatev, 2019). For example, researchers proposed the reflective model of prosociality to explain the reasons why people’s act was prosocial, stating that humans were born as creatures with non-moral and purely egoistic tendencies while prosocial behavior required exerting reflective control over this egoistic instinct (Metcalfe and Mischel, 1999; Stevens and Hauser, 2004). In addition, the human drive for cooperation and altruism was one of the most powerful forces shaping our society (Alos-Ferrer and Garagnani, 2018). With the continuous acceleration of globalization in today’s world, large-scale human cooperation was needed (Buchan et al., 2009). On average, people became more cooperative with age perhaps because experience taught them that cheating in many milieux was a losing strategy in the long run (Matsumoto et al., 2016), so honesty and cooperation were a desirable strategy.

Meanwhile, prosocial behavior was defined as a broad category of actions that were generally beneficial to other people, such as cooperating with, sharing resources with, and helping others (Penner et al., 2005). Moreover, trust was critical for building and maintaining relationships and active cooperation (Lewicki and Brinsfield, 2017). The ability of society to motivate its members to cooperate spontaneously characterized the extent of social cohesion within that society (Coleman, 1990; Roca and Helbing, 2011; Aksoy, 2019). In social dilemma (Olson, 1965; Marwell and Ames, 1979; Dawes, 1980; Taylor, 1987; Gambetta, 1988; Kollock, 1998), cooperation always resulted in a more excellent outcome for all individuals. In a two-person social dilemma (e.g., a prisoner’s dilemma), mutual cooperation always resulted in a greater outcome for each individual relative to mutual defection (Balliet and Van Lange, 2012). However, win-win requires transpositional consideration, which means achieving the optimal state of coordination between individuals and the collective. In the situation of win-win, both sides need mutual care and mutual benefit. Otherwise, neither side will get a good result.

However, few studies directly explored the psychological structure and its measurement of win-win. Up to now, researchers have developed the General Trust Scale, Adolescent Pro-Social Behavior Scale, Self-Consistency and Congruence Scale, Interpersonal Trust Scale, and Cooperative and Competitive Personality Scale (Rotter, 1967; Wang, 1994; Yamagishi and Yamagishi, 1994; Xie et al., 2006; Yang et al., 2016). On the one hand, these concepts of trust, prosocial behavior, harmony, and cooperation differ from win-win. Trust is “the extent to which a person is confident in, and willing to act on the basis of, the words, actions, and decisions of another” (McAllister, 1995). Trust is critical for building and maintaining relationships and for effectively working together. In cooperative behaviors, the individuals provide direct benefits to others at a cost to themselves, which we will call prosocial (Henrich and Henrich, 2006). In brief, trust and cooperation are important ways and means to achieve win-win results, and win-win is a very important prosocial behavior that has been neglected by psychological researchers. On the other hand, based on the probe into the win-win structure (Zhang and Zhang, 2020), the psychological dimensions of the win-win scale may be distinctive from these scales, such as General Trust Scale, Adolescent Pro-Social Behavior Scale, Self-Consistency, and Congruence Scale. In addition, cooperation is based on prosocial behavior and trust (Kramer, 1999; Penner et al., 2005), and the high levels of cooperation cannot be sustained merely based on the preferences and generalized trust that people carry around within them (Simpson and Willer, 2015). Only by taking the concept of win-win as the value orientation can all stakeholders maintain their survival and development in today’s pluralistic society. Therefore, developing a win-win scale has important implications for individuals and society.

To explore the dimensions of the public’s view on win-win, Zhang and Zhang (2020) made a preliminary study. Firstly, an open questionnaires survey (What do you think is necessary for people to achieve win-win value orientation? Or what are the characteristics of people pursuing win-win?) among 137 participants (60 males and 77 females ranged in age from 17 to 64 years; 16 participants had master’s degrees or above, 74 participants had bachelor’s degrees, 29 participants had associate bachelor’s degrees, and 17 participants had high school degrees or below) was conducted to develop a win-win’s characteristic words questionnaire. All the participants were provided informed consent, and they were asked to complete the questionnaire, which took about 5–10 min. They were informed about the confidentiality and anonymity of their responses prior to starting the questionnaires. Secondly, a cluster analysis of 332 participants (145 males and 187 females ranged in age from 16 to 57 years; 63 participants had master’s degrees or above, 172 participants had bachelor’s degrees, 62 participants had associate bachelor’s degrees, 33 participants had high school degrees or below, and two participants lacked educational background information) was conducted, and the results indicated that there were 29 characteristic words for win-win. The items with the top 10 average scores were honesty, respect for others, having a sense of the overall situation, team spirit, willingness to cooperate with others, pursuing mutual interests, understanding others, being good at transposition thinking, being good at listening, and being good at communicating with others. Moreover, the cluster analysis results showed that the public’s view on win-win could be divided into five dimensions: integrity, advancement, altruism, harmoniousness, and coordination. Based on the structural dimensions of win-win from Zhang and Zhang (2020), the present study intended to further develop the win-win scale as a useful assessment tool.

## Study 1

### Method

#### Participants

A total of 329 volunteer participants took part in the study, and 320 valid participants including 102 males and 218 females were obtained. Here, 201 participants were residents in urban areas, and 119 participants were residents in rural areas. In terms of education, 48 participants had master’s degrees or above, 223 participants had bachelor’s degrees, 35 participants had associate bachelor’s degrees, nine participants had high school degrees, and five participants had junior middle school degrees or below. Among them, 237 participants were full-time students, and 103 participants had working experience.

#### Materials

Based on previous research (Zhang and Zhang, 2020), 50 items were compiled to the initial questionnaire of win-win, reviewed by several graduate students and professional mentors of the psychology major to refine the items.

The initial questionnaire consisted of one polygraph question (“I have never cheated anyone.”) and nine reverse items. The questionnaire was a self-rated 5-point Likert scale (1 = “completely disagreed,” 2 = “relatively disagreed,” 3 = “uncertain,” 4 = “relatively agreed,” and 5 = “completely agreed”).

#### Procedure

Participants volunteered to complete the questionnaire on the website from March to April 2020. Participants were asked to choose the most suitable situation for themselves by reading the description of each sentence. SPSS 22.0 software was used for item analysis and exploratory factor analysis (EFA).

### Results

#### Item Analysis

Before the EFA, we used item analysis (Wu, 2010) for the preliminary selection of items, and the following two steps were performed: (1) Checking the data file to ensure that the data could be analyzed under the ordinary conditions in case of any error value or missing value; (2) Numerical conversion of the reverse items, recording and scoring the reverse items, and assigning new values to old ones.

After removing the polygraph item, 49 items were screened by item analysis. Firstly, the data were divided into high and low groups for independent-samples *t*-test by using the critical ratio method. The criteria for deletion were as follows: (1) The critical value was not significant (*p* > 0.05); (2) The *t* statistic of the difference between high and low item groups was lower than 3(*t* < 3). The results showed that item 35 (*t* = −0.279, *p* = 0.781), item 49 (*t* = 1.572, *p* = 0.118), item 24 (*t* = −2.808, *p* = 0.006), item 38 (*t* = 2.849, *p* = 0.005), and item 47 (*t* = 2.961, *p* = 0.003) were not up to standard, so they were deleted (Wu, 2010).

Furthermore, the correlation between the item score and the total score was calculated before the items were screened. Based on the Pearson correlation coefficient, the criteria for deletion were as follows: (1) The correlation between the items and the total scale was not significant (*p* > 0.05); (2) The correlation coefficient (*r*) between the item score and the total score was lower than 0.4. The result showed that item 9 (*r* = 0.395, *p* < 0.05), item 15 (*r* = 0.395, *p* < 0.05), item 24 (*r* = −0.185, *p* < 0.05), item 20 (*r* = 0.399, *p* < 0.05), item 28 (*r* = 0.269, *p* < 0.05), item 35 (*r* = 0.013, *p* = 0.811), item 38 (*r* = 0.132, *p* < 0.05), item 45 (*r* = 0.336, *p* < 0.05), item 47 (*r* = 0.202, *p* < 0.05), and item 49 (*r* = 0.067, *p* = 0.232) were not up to standard, so these items were deleted.

Then, the reliability coefficient was used to select the items. The internal consistency α coefficient of the 49 items was 0.922, indicating a good internal consistency of the scale. The deletion criteria were as follows: (1) The correlation coefficient between the modified item and the total score was lower than 0.45; (2) The internal consistency coefficient after item deletion would become larger. The results showed that total score correlation coefficients of the modified items in items 4, 9, 15, 16, 17, 20, 24, 28, 35, 38, 45, 47, 48, and 49 were all less than 0.45. If items 15, 24, 35, 38, 47, and 49 were deleted, the internal consistency coefficient would increase. Therefore, these items were deleted.

Finally, we used communalities and factor loading to screen the items by the principal component analysis (PCA) with maximum variance (Varimax) method. The deletion criteria were as follows: (1) The common value was lower than 0.2; (2) Factor loading was lower than 0.45. The results showed that common values for items 4 (0.176), 9 (0.153), 15 (0.023), 16 (0.195), 20 (0.094), 24 (0.079), 28 (0.027), 35 (0.007), 38 (0.001), 45 (0.053), 47 (0.008), 48 (0.196), and 49 (0.002) were not up to standard, so these items were deleted.

Combined with the above item analysis methods, 14 items were deleted and 35 items were retained.

#### Exploratory Factor Analysis

The Kaiser–Meyer–Olkin (KMO) value (KMO = 0.941) and Bartlett’s Test of Sphericity (χ*^2^* = 5142.646, *df* = 595, *p* < 0.001) showed that the items of this scale were appropriate for factor analysis. Besides, based on the analysis of the polygraph item that the higher score on this item indicated that participants failed to answer the question honestly (Yang and Zhao, 1990). In our study, 320 valid samples were retained, which met the minimum number of sample observations for each variable (Stevens, 2002). Moreover, PCA and Varimax were used to analyze 35 items. The items that did not meet the standard and theoretical expectation were deleted. The criteria for deletion were as follows: (1) The number [measures of sampling adequacy (MSA)] of sampling fitness for items was below 0.8; (2) The typical value was below 0.3; (3) The factor loading was lower than 0.45; (4) The item appeared in two or more factors at the same time; (5) There were only 1–2 items in the factors. As a result, items 8, 2, 5, 13, 19, 11, 42, 30, 36, and 41 were deleted.

Then, the remaining 25 items were used for EFA. Five factors emerged with eigenvalues larger than 1, with a cumulative variance interpretation rate of 57.54% (Table 1). All items’ communalities ranged from 0.45 to 0.67, and factor loadings ranged from 0.45 to 0.80. Factor 1 consisted of six items (23, 5, 1, 12, 32, and 43) named “integrity” that referred to honesty and trustworthiness. Factor 2 consisted of five items (18, 27, 46, 44, and 6) named “advancement” that meant the pursuit of excellence. Factor 3 consisted of six items (33, 34, 37, 25, 29, and 31) named “altruism” that implicated the action from the perspective of others’ interests. Factor 4 consisted of five items (10, 22, 7, 39, and 21) named “harmoniousness” that signified mutual respect and inclusion. Factor 5 consisted of three items (14, 3, and 40) named “coordination” that emphasized the consciousness of collectivity and the intention of cooperation.

**TABLE 1:** Factor analysis matrix of win-win.

Item	Factor loading					Communalities
	1	2	3	4	5	
23. I think people’s credit is very important.	0.799					0.672
5. I think honesty is the basis of win-win.	0.703					0.541
1. I treat people sincerely.	0.701					0.604
12. I agree that “no one can be accomplished without integrity.”	0.649					0.532
32. I actively fulfill my obligations.	0.631					0.598
43. I can keep my promise.	0.558					0.510
18. I can always achieve the goals I set for myself.		0.752				0.652
27. I can always concentrate on things.		0.709				0.631
46. I can learn professional knowledge quickly.		0.681				0.566
44. I always pursue excellence.		0.624				0.549
6. I always have an intense thirst for knowledge.		0.534				0.456
33. I will act in the interest of others.			0.757			0.636
34. I will take the initiative to work for the group.			0.680			0.674
37. It is worth to help others even if misunderstood.			0.605			0.522
25. I am willing to share my resources with others.			0.495			0.511
29. I think about the whole when I do somethings.			0.489			0.583
31. I often think from the perspective of others.			0.451			0.614
10. I can tolerate the shortcoming of others.				0.698		0.643
22. I can quickly reach an agreement with others.				0.607		0.564
7. I always get along well with others.				0.577		0.545
39. I make it a point to listen to the other person’s point of view.				0.569		0.524
21. I am happy to appreciate and learn the positive qualities of others.				0.465		0.460
14. I like to take part in group activities.					0.726	0.658
3. I often solve problems with my friends.					0.584	0.560
40. I often discuss problems with others.					0.566	0.579
Eigenvalues	9.063	1.768	1.301	1.252	1.002	
Contribution rate (%)	36.251	7.071	5.203	5.007	4.006	
Cumulative contribution rate (%)	36.251	43.323	48.525	53.532	57.538	

*Extract: principal component analysis (PCA); Rotation: Varimax.*

## Study 2

Study 2 used another sample to conduct confirmatory factor analysis (CFA) of the first-order and second-order models. This goal was to provide initial evidence of the win-win structure.

### Method

#### Participants

A total of 270 participants took part in Study 2 and 250 valid questionnaires were received, with an effective rate of 92.6%. There were 59 males and 191 females. Here, 163 participants were residents in urban areas, and 87 participants were residents in rural areas. In terms of education, 18 participants had master’s degrees or above, 168 participants had bachelor’s degrees, 42 participants had associate bachelor’s degrees, 16 participants had high school degrees, and six participants had junior middle school degrees or below. Among them, 157 participants were full-time students, and 93 participants had working experience.

#### Materials

Based on 25 items identified by EFA in Study 1, these items in Study 2 were recompiled. The scale items were arranged from simple to complicated, and the order of dimension items was randomly distributed. Participants were asked to read each sentence’s description and then choose the most suitable option for their actual situation (1 = “completely disagreed,” 2 = “relatively disagreed,” 3 = “uncertain,” 4 = “relatively agreed,” and 5 = “completely agreed”).

#### Procedure

The sample data used for CFA were collected from April to May 2020. The participants filled in the questionnaire through the online website. AMOS 22.0 software was used to analyze the five-factor model. The maximum likelihood method was chosen for the model parameter estimation to explore the relationship between items and latent variables.

### Result

#### Confirmatory Factor Analysis of the First-Order Model

CFA aimed to identify the goodness of fit between a model and obtained data (Sumbuloglu and Akdag, 2009). Given the five-factor solution identified in EFA, we drew the model diagram according to the 25 items and five factors obtained from EFA. CFA of the first-order model was carried out, and nine items (a1, a17, a23, a4, a9, a15, a20, a6, and a10) were deleted because of the lower modification indexes (MIs) for these items (Hau et al., 2005; Jiao et al., 2019).

The model fitting index was selected with degrees of freedom, root mean square error of approximation (RMSEA), root mean square residual (RMR), standardized RMR (SRMR), Comparative Fit Index (CFI), Goodness of Fit Index (GFI), and Tucker–Lewis Index (TLI). Tabachnick and Fidell (2007) and Kline (2005) recommended the value of χ*^2^/df* in ranges of 1 to 2 or 1 to 3 as an indicator of a good fit. CFI, GFI, and TLI’s recommended values should be greater than 0.90, RMSEA and RMR are less than 0.08 for a good model fit (Hu and Bentler, 1999). The results of the goodness-of-fit were as follows: χ*^2/^df* = 2.112; RMSEA was 0.067; both RMR and SRMR were lower than 0.05; CFI, GFI, and TLI were higher than 0.90. The results indicated that the model was within the acceptable fit indexes (Table 2).

**TABLE 2:** Goodness-of-fit indexes for five-factor model using the first-order CFA.

Indexes	*χ ^2^*	*df*	RMSEA	RMR	SRMR	CFI	GFI	TLI
First-order	198.57	94	0.067	0.038	0.044	0.957	0.911	0.945

*RMSEA, Root Mean Square Error of Approximation; RMR, Root Mean Square Residual; SRMR, Standardized Root Mean Square Residual; CFI, Comparative Fit Index; GFI, Goodness of Fit Index; TLI, Tucker-Lewis Index.*

**FIGURE 1:**
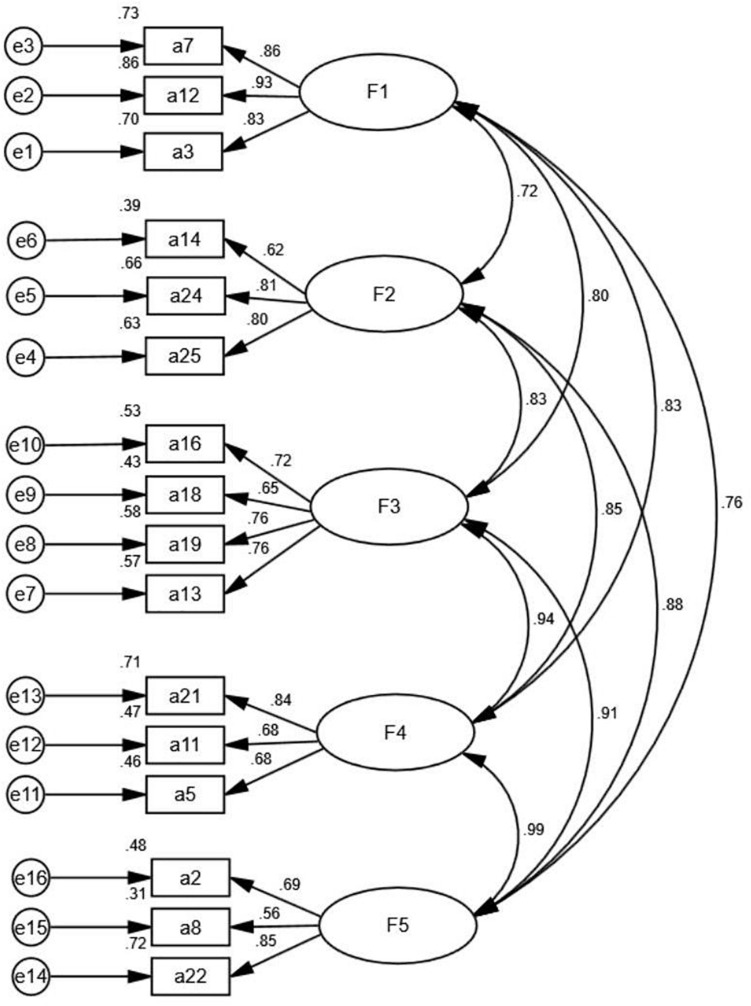
The first-order CFA model of win-win.

The correlation coefficients among the five factors (Figure 1) suggested that the first-order factor constructs were influenced by a higher-order latent trait (Wu, 2009). Thus, this study carried out CFA of the second-order model.

#### Confirmatory Factor Analysis of the Second-Order Model

CFA of the second-order model was conducted according to the previous research (Bolton, 1980; Chu, 2008; Wu, 2009; Black et al., 2015). In the analysis of second-order factor, it was assumed that the extracted latent variables in the preceding stage were present. Thus, the second-order factor analysis represented the more general concepts at secondary and upper levels (Gatignon, 2014). The second-order factor analysis was used to examine whether or not all the factors fitted the general concept of win-win (Sharif Nia et al., 2019).

**FIGURE 2:**
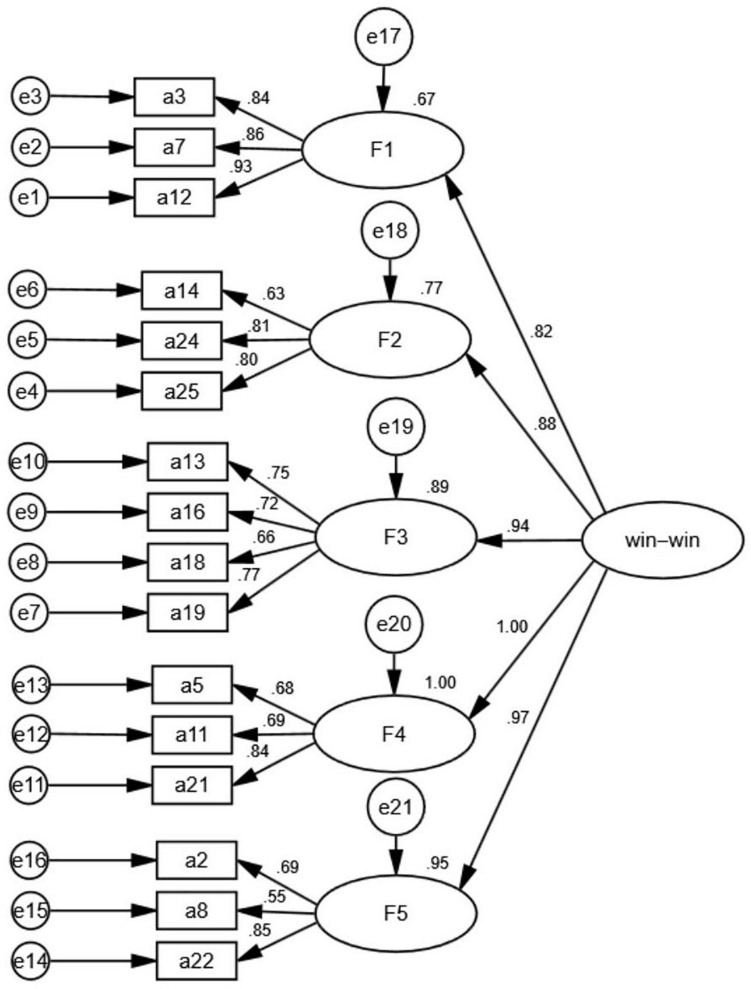
The second-order CFA model of win-win.

The results showed that χ*^2^*/*df* = 2.1; RMSEA was 0.066; both RMR and SRMR were lower than 0.05; CFI, GFI, and TLI were higher than 0.90 (Table 3). The structural model with standardized parameter estimates was shown in Figure 2. The factor loadings of the five factors were 0.82, 0.88, 0.94, 1.00, and 0.97. The internal consistency reliability coefficients of the five factors were 0.67, 0.77, 0.89, 1.00, and 0.95. The above results indicated that the overall assessment of the criteria for model fit was acceptable.

**TABLE 3:** Goodness-of-fit indexes for five-factor model using the second-order CFA.

Indexes	*χ ^2^*	*df*	RMSEA	RMR	SRMR	CFI	GFI	TLI
Second-order	207.92	99	0.066	0.039	0.045	0.955	0.907	0.946

### Internal Consistency Reliability

The Cronbach’s alpha of factors 1, 2, 3, 4, and 5 were 0.907, 0.780, 0.816, 0.786, and 0.740, respectively. The Cronbach’s alpha of the whole scale was 0.959, indicating high internal consistency reliability.

### Convergent Validity

The scale’s Average Variance Extracted (AVE) values and Construct Reliability (CR) values were shown in Table 4. The results showed that the AVE values of all the factors were above 0.5, the CR values were above 0.7, and the factor loadings of the items were above 0.5, indicating a good convergence validity for the model by CFA.

**TABLE 4:** The convergent validity of win-win.

Factor	Item	Estimate	AVE	CR
F1	a3.I think honesty is the basis of win-win.	0.835	0.764	0.907
	a7.I agree that “no one can be accomplished without integrity.”	0.856		
	a12.I think people’s credit is very important.	0.929		
F2	a14.I can always concentrate on things.	0.622	0.561	0.791
	a24.I always pursue excellence.	0.814		
	a25.I can learn professional knowledge quickly.	0.796		
F3	a13.I am willing to share my resources with others.	0.755	0.527	0.816
	a16.I often think from the perspective of others.	0.725		
	a18.I will act in the interest of others.	0.655		
	a19.I will take the initiative to work for the group.	0.763		
F4	a5.I always get along well with others.	0.678	0.547	0.782
	a11.I can quickly reach an agreement with others.	0.683		
	a21.I make it a point to listen to the other person’s point of view.	0.845		
F5	a2. I often solve problems with my friends.	0.689	0.502	0.746
	a8.I like to take part in group activities.	0.558		
	a22.I often discuss problems with others.	0.848		

## Discussion

The goal of the present study was to develop and validate a win-win scale. Our preliminary research (Zhang and Zhang, 2020) firstly employed an open questionnaire survey, and 29 characteristic words for win-win were obtained. Then, a cluster analysis study was carried out based on a questionnaire investigation using a 5-point Likert score, and five win-win structures were obtained. Based on the pilot research (Zhang and Zhang, 2020), 50 items were formed to explore the psychological structure of win-win in the current study. Using item analysis, EFA, and CFA, 25 items were remained and five dimensions (integrity, advancement, altruism, harmoniousness, and coordination) were constructed. To ensure the model’s simplicity in the CFA phase, nine items were deleted according to the MI and fitting degree indexes. Finally, 16 items were obtained, and the five dimensions’ model of win-win fit well. The reliability and validity of our scale met the criteria. Therefore, our study verified the results of Zhang and Zhang (2020) and contributed to measuring win-win for theoretical and practical application.

Our results revealed that the win-win scale contained five dimensions including integrity, advancement, altruism, harmoniousness, and coordination. Integrity is one of the primary bonds of interpersonal communication (Zhang and Zhong, 2017). Only with integrity can we achieve win-win. Advancement is presented as the desire for making progress or a tendency to develop. If people have high requirements for themselves, then they will be able to face various conflicts and problems positively (Vera et al., 2004; Desivilya and Eizen, 2005), which can be beneficial to realize win-win. Harmoniousness affects people’s social communication and interaction (Gabrenya and Hwang, 1996). Harmoniousness does not mean avoiding conflicts blindly; it is defined as the combination and unified coexistence of different things. Only mutual respect and inclusion can attain win-win. The core content of altruism is transpositional consideration rather than paying attention to self without others’ thoughts. If one only blindly cares about one’s self-interests, this will result in a lousy ending. A better ending of win-win requires common development and taking care of others. The last dimension is coordination, reflecting win-win for two or more people working together rather than only one person to struggle. To sum up, our results demonstrated that the five dimensions were indispensable for win-win.

Win-win is the realization of self-interest and mutual benefit. The stakeholders stand to gain from cooperation and lose from confrontation. Win-win was regarded a value orientation that derived from Chinese traditional culture (Sima, 1956; Zhang, 1996) and generalized across cultural settings. “The doctrine of the mean” and “Great harmony” embody the essential characteristics of win-win coexistence and mutual prosperity. Win-win is the best state that different stakeholders can achieve. The current study was the first to develop a win-win scale based on the research on cooperation, trust, and prosocial behavior (Smith, 2015; Duijf, 2018; Zlatev, 2019), and it expanded existing research. Moreover, the present study was of great value in promoting the harmonious development of humans and providing new perspectives for creating a community of shared future.

## Limitations and Future Directions

The present study is still in the preliminary stage in the psychological discipline. There are still some limitations. Firstly, there may be sampling bias. There was an imbalance between gender and education in the sample size in this study. Although previous studies demonstrated measurement invariance across grades and genders for some scales (e.g., Harter, 1982; Cheung and Rensvold, 2002; Kim et al., 2019), the unbalanced numbers of participants on different gender/education levels may still affect our results. Future studies should obtain a balanced sample to further test the win-win scale. Secondly, there may be a social desirability effect on self-administered questionnaire. Future studies could use other research methods (e.g., field research) to cross-validate our results. Lastly, it also would be important to investigate whether there is a difference in win-win values among different groups, which is helpful to verify the reliability and validity of the current scale.

## Conclusion

The win-win scale contained five dimensions including integrity, advancement, altruism, harmoniousness, and coordination. It proved to be a reliable and valid tool for measuring win-win.

## Data Availability Statement

The original contributions presented in the study are included in the article/Supplementary Material, further inquiries can be directed to the corresponding author.

## Ethics Statement

The studies involving human participants were reviewed and approved by The Ethics Committee of Henan University. The participants provided their written informed consent to participate in this study.

## Author Contributions

FZ contributed to the conception and design of the study. SZ involved in implementing the study, data collection, and statistical analysis. SZ and XZ wrote the first draft of the manuscript. XZ polished and revised the draft. All authors contributed to the article and approved the submitted version.

## Conflict of Interest

The authors declare that the research was conducted in the absence of any commercial or financial relationships that could be construed as a potential conflict of interest.
