# Corrigendum: Development and Validation of the Win-Win Scale

**DOI:** 10.3389/fpsyg.2021.719890

**Published:** 2021-08-23

**Authors:** Shan Zhang, Xinlei Zang, Feng Zhang

**Affiliations:** Institute of Psychology and Behavior, Henan University, Kaifeng, China

**Keywords:** win-win, scale development, factor analysis, reliability, validity

In the original article, there were errors. **The descriptions of these sentences were not sufficiently clear**.

(1) A correction has been made to ^**^Introduction section, Paragraph one, the first sentence^*^^*^:

**The original sentence read: “To balance between what is best for individuals and what is best for the collective ones has long been central to sociology and other social sciences (Simpson and Willer**, **2015).” The corrected sentence is below:**

**Research on the balance between what is best for individuals and what is best for the collective ones has long been central to sociology and other social sciences (Simpson and Willer**, **2015**).

(2) A correction has been made to ^**^Introduction section, Paragraph two, the fifth sentence^**^. The original sentence read: “The importance of win-win cooperation is also reflected in “if two people reach an agreement, they can overcome all difficulties” in *The Book of Changes* (Tze, 2011) and “one is liable to fail, and if there are many, it is hard to be defeated” in *History as a Mirror* (Sima, 1956).” The corrected sentence is below:

**The importance of win-win cooperation is also reflected in “if two people reach an agreement, they can overcome all difficulties” in*****The Book of Changes*** (**Tze**, **2011) and “one is liable to fail, and if there are many people, it is hard to be defeated” in** ***History as a Mirror*** (**Sima**, **1956)**.

(3) A correction has been made to ^**^Study 1, Method, Participants, Paragraph one, the third sentence^**^. The original sentence read: “In terms of education, 48 participants had master's degrees or above, 223 participants had bachelor's degrees, 35 participants had associate bachelor's degrees, nine participants had high school degrees, and five participants had a junior middle school degrees or below.” The corrected sentence is below:

**In terms of education, 48 participants had master's degrees or above, 223 participants had bachelor's degrees, 35 participants had associate bachelor's degrees, nine participants had high school degrees, and five participants had junior middle school degrees or below**.

(4) A correction has been made to ^**^Study 1, Results, Item Analysis, Paragraph four, the last sentence^**^. The original sentence read: “Therefore, there was a difference between what we wanted to measure and measured, so these items were deleted.” The corrected sentence is below:

**Therefore, these items were deleted**.

(5) A correction has been made to ^**^Discussion section, Paragraph one, the eighth sentence^**^. The original sentence read: “The reliability and validity of our scale caught the criteria.” The corrected sentence is below:

**The reliability and validity of our scale met the criteria**.

The authors apologize for these errors and state that they do not change the scientific conclusions of the article in any way. The original article has been updated.

## Publisher's Note

All claims expressed in this article are solely those of the authors and do not necessarily represent those of their affiliated organizations, or those of the publisher, the editors and the reviewers. Any product that may be evaluated in this article, or claim that may be made by its manufacturer, is not guaranteed or endorsed by the publisher.
