# Supplementary material for: Development and Validation of the Win-Win Scale
Source: Front Psychol. 2021 May 21;12:657015. doi: 10.3389/fpsyg.2021.657015 (PMC8175639; doi:10.3389/fpsyg.2021.657015)
Supplement: Supplementary file 1 [file Data_Sheet_1.zip › data sheet 1/Questionnaire/1.Questionnaire EFA.docx]

| Description | Completely disagreed | | Relatively disagreed | | Uncertain | | Relatively agreed | | | Completely agreed |
| --- | --- | --- | --- | --- | --- | --- | --- | --- | --- | --- |
|  | | | | | | | | | | |
| 1. I treat people sincerely. |  | |  | |  | |  | | |  |
| 2. I often help others. |  | |  | |  | |  | | |  |
| 3. I often solve problems with my friends. |  | |  | |  | |  | | |  |
| 4. I often take part in voluntary activities. |  | |  | |  | |  | | |  |
| 5. I think honesty is the basis of win-win. |  | |  | |  | |  | | |  |
| 6. I always have an intense thirst for knowledge. |  | |  | |  | |  | | |  |
| 7. I always get along well with others. |  | |  | |  | |  | | |  |
| 8. I can express my ideas clearly. |  | |  | |  | |  | | |  |
| 9. I take the initiative in interpersonal communication. |  | |  | |  | |  | | |  |
| 10. I can tolerate the shortcoming of others. |  | |  | |  | |  | | |  |
|  | | | | | | | | | | |
| 11. I can solve problems quickly. |  | |  | |  | |  | | |  |
| 12. I agree that "no one can be accomplished without integrity. " |  | |  | |  | |  | | |  |
| 13. I would like to donate to charity. |  | |  | |  | |  | | |  |
| 14. I like to take part in group activities. |  | |  | |  | |  | | |  |
| 15. I often do things that violate the rules. |  | |  | |  | |  | | |  |
| 16. I believe in "take a step back when in trouble". | |  | |  | |  | |  |  | |
| 17. I can quickly work with unfamiliar people. | |  | |  | |  | |  |  | |
| 18. I can always achieve the goals I set for myself. | |  | |  | |  | |  |  | |
| 19. I act in the interest of others. | |  | |  | |  | |  |  | |
| 20. I often blame others when I fail. | |  | |  | |  | |  |  | |
|  | | | | | | | | | | |
| 21. I am happy to appreciate and learn the positive qualities of others. | |  | |  | |  | |  |  | |
| 22. I can quickly reach an agreement with others. | |  | |  | |  | |  |  | |
| 23. I think people's credit is very important. | |  | |  | |  | |  |  | |
| 24. I often insist on my own opinion. | |  | |  | |  | |  |  | |
| 25. I am willing to share my resources with others. | |  | |  | |  | |  |  | |
| 26. I have never cheated anyone. | |  | |  | |  | |  |  | |
| 27. I can always concentrate on things. | |  | |  | |  | |  |  | |
| 28. I often have conflicts with others. | |  | |  | |  | |  |  | |
| 29. I think about the whole when I do somethings. | |  | |  | |  | |  |  | |
| 30. I pay more attention to improving my quality. | |  | |  | |  | |  |  | |
|  | | | | | | | | | | |
| 31. I often think from the perspective of others. | |  | |  | |  | |  |  | |
| 32. I actively fulfill my obligations. | |  | |  | |  | |  |  | |
| 33. I will act in the interest of others. | |  | |  | |  | |  |  | |
| 34. I will take the initiative to work for the group. | |  | |  | |  | |  |  | |
| 35. I cannot accept strange ideas from others. | |  | |  | |  | |  |  | |
| 36. I can adjust my mind quickly. | |  | |  | |  | |  |  | |
| 37. It is worth to help others even if misunderstood. | |  | |  | |  | |  |  | |
| 38. I think most people cheat. | |  | |  | |  | |  |  | |
| 39. I make it a point to listen to the other person's point of view. | |  | |  | |  | |  |  | |
| 40. I often discuss problems with others. | |  | |  | |  | |  |  | |
|  | | | | | | | | | | |
| 41. When others are in trouble, I often lend them a hand. | |  | |  | |  | |  |  | |
| 42. I think collaboration is what gets things done. | |  | |  | |  | |  |  | |
| 43. I can keep my promise. | |  | |  | |  | |  |  | |
| 44. I always pursue excellence. | |  | |  | |  | |  |  | |
| 45. I will do things at the expense of others. | |  | |  | |  | |  |  | |
| 46. I can learn professional knowledge quickly. | |  | |  | |  | |  |  | |
| 47. I do not like to discuss problems with others, even if they are troublesome. | |  | |  | |  | |  |  | |
| 48. I trust the people around me. | |  | |  | |  | |  |  | |
| 49. I don't like to work with people whose values don't coincide with mine. | |  | |  | |  | |  |  | |
| 50. I think most people stick to their faith. | |  | |  | |  | |  |  | |

| 描 述 | 完全  不符合 | 比较  不符合 | 不确定 | | 比较  符合 | 完全  符合 | |
| --- | --- | --- | --- | --- | --- | --- | --- |
|  | | | | | | | |
| 1.我待人真诚。 |  |  |  | |  |  | |
| 2.我经常帮助他人。 |  |  |  | |  |  | |
| 3.我经常和朋友一起解决问题。 |  |  |  | |  |  | |
| 4.我经常参加志愿活动。 |  |  |  | |  |  | |
| 5.我认为诚信是共赢的基础。 |  |  |  | |  |  | |
| 6.我总有强烈的求知欲。 |  |  |  | |  |  | |
| 7.我总是能和别人相处得很愉快。 |  |  |  | |  |  | |
| 8.我能清晰地表达自己的观点。 |  |  |  | |  |  | |
| 9.我在人际交往中比较主动。 |  |  |  | |  |  | |
| 10.我能包容他人的缺点。 |  |  |  | |  |  | |
|  | | | | | | | |
| 11.我能很快解决遇到的问题。 |  |  |  | |  |  | |
| 12.我认同“人无信不立”。 |  |  |  | |  |  | |
| 13.我愿意为慈善献爱心。 |  |  |  | |  |  | |
| 14.我喜欢参加团体活动。 |  |  |  | |  |  | |
| 15.我常做一些违反规则的事。 |  |  |  | |  |  | |
| 16.我相信“退一步，海阔天空”。 |  |  | |  |  | |  |
| 17.我能很快和不熟悉的人合作。 |  |  | |  |  | |  |
| 18.我总能实现设定的目标。 |  |  | |  |  | |  |
| 19.我做事会考虑他人的利益。 |  |  | |  |  | |  |
| 20.我在失败时经常抱怨别人。 |  |  | |  |  | |  |
|  | | | | | | | |
| 21.我乐于欣赏并学习他人的积极品质。 |  |  | |  |  | |  |
| 22.我能很快和别人达成共识。 |  |  | |  |  | |  |
| 23.我认为人的信用是非常重要的。 |  |  | |  |  | |  |
| 24.我经常坚持我自己的意见。 |  |  | |  |  | |  |
| 25.我愿意与他人共享资源。 |  |  | |  |  | |  |
| 26.我从来没有欺骗过他人。 |  |  | |  |  | |  |
| 27.我总能集中精力做事。 |  |  | |  |  | |  |
| 28.我经常和他人发生矛盾与冲突。 |  |  | |  |  | |  |
| 29.我做事时会通盘考虑整体利益。 |  |  | |  |  | |  |
| 30.我非常注重提高自身素质。 |  |  | |  |  | |  |
|  | | | | | | | |
| 31.我经常站在他人角度思考问题。 |  |  | |  |  | |  |
| 32.我积极履行自己应承担的义务。 |  |  | |  |  | |  |
| 33.我会以他人利益为出发点来行事。 |  |  | |  |  | |  |
| 34.我会为了集体而主动做事。 |  |  | |  |  | |  |
| 35.我不能接受别人奇怪的想法。 |  |  | |  |  | |  |
| 36.我可以快速调整好自己的心态。 |  |  | |  |  | |  |
| 37.即使被误解，帮助别人也是值得的。 |  |  | |  |  | |  |
| 38.我认为大多数人都会欺骗他人。 |  |  | |  |  | |  |
| 39.我重视倾听他人的观点。 |  |  | |  |  | |  |
| 40.我经常和别人一起讨论问题。 |  |  | |  |  | |  |
|  | | | | | | | |
| 41.在别人遇到困难时，我经常伸出援手。 |  |  | |  |  | |  |
| 42.我认为彼此协作才能把事情做好。 |  |  | |  |  | |  |
| 43.我能遵守自己的承诺。 |  |  | |  |  | |  |
| 44.我总是不断追求卓越。 |  |  | |  |  | |  |
| 45.我会做损人利己的事情。 |  |  | |  |  | |  |
| 46.我能快速学习专业知识。 |  |  | |  |  | |  |
| 47.即使问题很麻烦，我也不喜欢和别人一起商量。 |  |  | |  |  | |  |
| 48.我很信任我周围的人。 |  |  | |  |  | |  |
| 49.我不喜欢与我价值观不一致的人共事。 |  |  | |  |  | |  |
| 50.我认为大多数人能坚守诚信。 |  |  | |  |  | |  |
