# Supplementary material for: Development and Validation of the Win-Win Scale
Source: Front Psychol. 2021 May 21;12:657015. doi: 10.3389/fpsyg.2021.657015 (PMC8175639; doi:10.3389/fpsyg.2021.657015)
Supplement: Supplementary file 1 [file Data_Sheet_1.zip › data sheet 1/Questionnaire/2.Questionnaire CFA.docx]

| Description | Completely disagreed | | Relatively disagreed | Uncertain | Relatively agreed | Completely agreed |
| --- | --- | --- | --- | --- | --- | --- |
|  | | | | | | |
| 1. I treat people sincerely. |  | |  |  |  |  |
| 2. I often solve problems with my friends. |  | |  |  |  |  |
| 3. I think honesty is the basis of win-win. |  | |  |  |  |  |
| 4. I always have an intense thirst for knowledge. |  | |  |  |  |  |
| 5. I always get along well with others. |  | |  |  |  |  |
| 6. I can tolerate the shortcoming of others. |  | |  |  |  |  |
| 7. I agree that “no one can be accomplished without integrity. " |  | |  |  |  |  |
| 8. I like to take part in group activities. |  | |  |  |  |  |
| 9. I can always achieve the goals I set for myself. |  | |  |  |  |  |
| 10. I am happy to appreciate and learn the positive qualities of others. |  | |  |  |  |  |
|  | | | | | | |
| 11. I can quickly reach an agreement with others. |  | |  |  |  |  |
| 12. I think people’s credit is very important. |  | |  |  |  |  |
| 13. I am willing to share my resources with others. |  | |  |  |  |  |
| 14. I can always concentrate on things. |  | |  |  |  |  |
| 15. I think about the whole when I do somethings. |  | |  |  |  |  |
| 16. I often think from the perspective of others. | |  |  |  |  |  |
| 17. I actively fulfill my obligations. | |  |  |  |  |  |
| 18. I will act in the interest of others. | |  |  |  |  |  |
| 19. I will take the initiative to work for the group. | |  |  |  |  |  |
| 20. It is worth to help others even if misunderstood. | |  |  |  |  |  |
|  | | | | | | |
| 21. I make it a point to listen to the other person’s point of view. | |  |  |  |  |  |
| 22. I often discuss problems with others. | |  |  |  |  |  |
| 23. I can keep my promise. | |  |  |  |  |  |
| 24. I always pursue excellence. | |  |  |  |  |  |
| 25. I can learn professional knowledge quickly. | |  |  |  |  |  |

| 描 述 | 完全  不符合 | 比较  不符合 | | 不确定 | | 比较  符合 | 完全  符合 | |
| --- | --- | --- | --- | --- | --- | --- | --- | --- |
|  | | | | | | | | |
| 1.我待人真诚。 |  |  | |  | |  |  | |
| 2.我经常和朋友一起解决问题。 |  |  | |  | |  |  | |
| 3.我认为诚信是共赢的基础。 |  |  | |  | |  |  | |
| 4.我总有强烈的求知欲。 |  |  | |  | |  |  | |
| 5.我总是能和别人相处得很愉快。 |  |  | |  | |  |  | |
| 6.我能包容他人的缺点。 |  |  | |  | |  |  | |
| 7.我认同“人无信不立”。 |  |  | |  | |  |  | |
| 8.我喜欢参加团体活动。 |  |  | |  | |  |  | |
| 9.我总能实现设定的目标。 |  |  | |  | |  |  | |
| 10.我乐于欣赏并学习他人的积极品质。 |  |  | |  | |  |  | |
|  | | | | | | | | |
| 11.我能很快和别人达成共识。 |  |  | |  | |  |  | |
| 12.我认为人的信用是非常重要的。 |  |  | |  | |  |  | |
| 13.我愿意与他人共享资源。 |  |  | |  | |  |  | |
| 14.我总能集中精力做事。 |  |  | |  | |  |  | |
| 15.我做事时会通盘考虑整体利益。 |  |  | |  | |  |  | |
| 16.我经常站在他人角度思考问题。 |  | |  | |  |  | |  |
| 17.我积极履行自己应承担的义务。 |  | |  | |  |  | |  |
| 18.我会以他人利益为出发点来行事。 |  | |  | |  |  | |  |
| 19.我会为了集体而主动做事。 |  | |  | |  |  | |  |
| 20.即使被误解，帮助别人也是值得的。 |  | |  | |  |  | |  |
|  | | | | | | | | |
| 21.我重视倾听他人的观点。 |  | |  | |  |  | |  |
| 22.我经常和别人一起讨论问题。 |  | |  | |  |  | |  |
| 23.我能遵守自己的承诺。 |  | |  | |  |  | |  |
| 24.我总是不断追求卓越。 |  | |  | |  |  | |  |
| 25.我能快速学习专业知识。 |  | |  | |  |  | |  |
